# Smartphone-Based Platform for Affect Monitoring through Flexibly Managed Experience Sampling Methods

**DOI:** 10.3390/s19153430

**Published:** 2019-08-05

**Authors:** Carlos Bailon, Miguel Damas, Hector Pomares, Daniel Sanabria, Pandelis Perakakis, Carmen Goicoechea, Oresti Banos

**Affiliations:** 1 Department of Computer Architecture and Technology, CITIC-UGR Research Center, University of Granada, E18071 Granada, Spain; 2 Mind, Brain & Behavior Research Center, University of Granada, E18071 Granada, Spain; 3 Department of Psychology, Loyola University Andalusia, E41014 Seville, Spain

**Keywords:** affective state, mood, flexible experience sampling, flexible esm, context, mHealth, mobile sensing, smartphone

## Abstract

The identification of daily life events that trigger significant changes on our affective state has become a fundamental task in emotional research. To achieve it, the affective states must be assessed in real-time, along with situational information that could contextualize the affective data acquired. However, the objective monitoring of the affective states and the context is still in an early stage. Mobile technologies can help to achieve this task providing immediate and objective data of the users’ context and facilitating the assessment of their affective states. Previous works have developed mobile apps for monitoring affective states and context, but they use a fixed methodology which does not allow for making changes based on the progress of the study. This work presents a multimodal platform which leverages the potential of the smartphone sensors and the Experience Sampling Methods (ESM) to provide a continuous monitoring of the affective states and the context in an ubiquitous way. The platform integrates several elements aimed to expedite the real-time management of the ESM questionnaires. In order to show the potential of the platform, and evaluate its usability and its suitability for real-time assessment of affective states, a pilot study has been conducted. The results demonstrate an excellent usability level and a good acceptance from the users and the specialists that conducted the study, and lead to some suggestions for improving the data quality of mobile context-aware ESM-based systems.

## 1. Introduction

Affective states, also referred to as mood [1], are an inherent part of our daily life. They provide our emotional context, shaping the background of everything we experience and do [2]. Furthermore, they play an active role when determining both the cognition content and the cognitive processes. For that reason, there is a strong evidence that affective states are highly related to some clinical disorders as depression, bipolarity or anxiety [3,4]. However, the changes on affective states also influence the cognition and social behavior of individuals who do not suffer from emotional disorders.

The affective states of an individual do not remain constant, they fluctuate over time. They are strongly influenced by the situations which individuals find themselves in, i.e., the context surrounding them [5]. These fluctuations may be caused by situational variables (location, social interaction or weather), as well as internal variables (sleep patterns, physical activity levels or stress). Therefore, identifying daily life events or situations that could lead to a change in the individual’s affective profile has raised as a fundamental task in emotion research [3,6], as it can help us not only to model the evolution of affective states during the daily life, but also to understand the source of these changes. In order to accomplish this task, both affective state and context must be monitored continuously during the course of the day, so that the fluctuations of the affective state are registered along with information about what is happening around the subject. Nevertheless, a significant proportion of the analyses performed during the last years do not take into consideration the daily context data when evaluating the affective state [7,8]. Even when the context is considered, some of them make use of self-reported information, leading to errors due to recall and subjective interpretation of the context [4,9].

With the recent emergence of mobile technologies, a new breed of intelligent mechanisms have raised to ubiquitously monitor affective processes and daily life context [10,11]. In particular, smartphones are at the leading edge because of their richness in terms of sensors and computing resources, and their widespread use among every segment of the population. As we carry the smartphone with us almost every time, these devices offer the potential to measure our context and behavior continuously, objectively, and with a minimal effort for the user. Moreover, assessing mood and context through the smartphone prevent individuals from carrying around extra devices that could modify their behavior. For those reasons, they have powered real-time monitoring techniques, such as the Experience Sampling Method (ESM) [12,13], one of the most commonly used methods for recording affective data.

In view of the present challenges of mood variability assessment, in [14] we presented the prototype of an integrated, multimodal platform that supports context-aware mobile ESM-reporting of the affective states during daily life. The present work completes and extends the implementation of this platform, describes it in detail, and assesses its validity and usability through a preliminary study. The proposed system is aimed to ubiquitously monitor the fluctuation of the affective states of an individual over time, and to perform an objective acquisition of its context through the sensors available in the smartphone. Moreover, the platform integrates the flexible management of the ESM questionnaires and smartphone sensor setup, the acquisition of the affect and context data through the smartphone, and the storage, visualization and processing of the gathered data. Section 2 presents an overview of the state-of-the-art in affective assessment through mobile phones. The platform and its operation are described in Section 3. The usability analysis and the evaluation of the platform are depicted in Section 4. The results of the evaluation are discussed in Section 5, and final conclusions and remarks are summarized in Section 6.

## 2. Related Work

**Figure 1:**
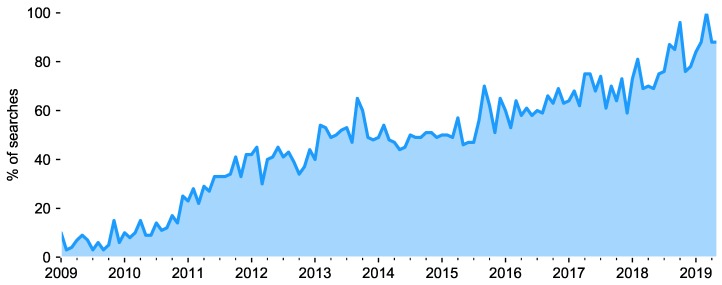
Number of searches of “mood app” during the last 10 years. Values expressed in percentage relative to the total amount of searches on that topic. Source: Google Trends [17].

In recent years, the massive presence of smartphones among the population has raised mobile technology as a resource for ubiquitous personal sensing. As people carry them almost everywhere -the global mobile internet usage stands at 76% [15], smartphones can be easily utilized in health and affective research to assess experiences during daily life [16]. Their user-friendliness make people feel comfortable using their own device, making research studies less intrusive. In our society, the interest in tracking our mood for a better understanding of it has also been growing during the last years. Figure 1 shows the worldwide search trend of the concept “mood app”. It shows that, during the last decade, people’s interest in this sort of apps has been increasing, and it still does. The values, depicted in percentages, reflect the amount of searches done, relative to the total of searches on that topic done on Google over time. It is also worth mentioning that this trend only refers to searches in English.

Within the scope of scientific research, the number of articles regarding this topic has also considerably increased. A systematic search through the Scopus and Web of Science scientific databases of the keywords “(*mood* OR *affective* OR *emotion*) AND (*monitoring* OR *assessment* OR (*evaluation*)) AND (*mobile* OR *smartphone*)” has been performed. The results obtained show that before year 2007, almost no article under this topic was indexed and, from this year on, the number of records has been increasing drastically, especially during the last decade. This finding, along with the fact that the total amount of articles found is not very high, proves that there is a good opportunity for the development of systems for the support of context-aware monitoring of affective states.

Several examples of affective states monitoring during the daily life using mobile technologies can be found in the literature, some of them leading to the development of specific monitoring apps. The vast majority of them ask individuals to assess their mood several times per day through self-reports, collected with the smartphone using the ESM technique. For example, in [18] a computerized mobile phone method is used to assess the participants’ mood in daily life via short phone calls. Other similar examples can also be found [3,16,19]. However, these studies do not consider capturing the context surrounding the subjects, thus lacking important information that could have an impact on the mood data and provide a better understanding of how it fluctuates. Mobile systems aimed at including the capture of context are also present in the literature. MoodPrism [20] uses mobile ESM questionnaires delivered at the smartphone in order to assess the emotional state. The application also collects data from social networks and the music listened to infer the mood through text analysis. Some extra context features were also gathered by means of self-reports in the app. MoodScope and MoodMiner [21,22] have also leveraged the potential of built-in mobile phone sensors to objectively capture context and predict mood. Situational features such as location, social interaction, ambient light and noise, and physical activity are inferred from the smartphone sensors to contextualize the affective data acquired. Similar but also recent examples are [23,24]. These works have achieved encouraging results, and prove that this methodology is technically feasible for sensing mood. Nevertheless, these works present mobile apps with a fixed methodology of data acquisition. The content of the ESM questionnaires, their scheduling, and the smartphone sensor configuration are defined prior to the beginning of the data collection. This approach does not allow for making changes on the methodology based on the issues encountered during the progress of the study, which could be useful to improve certain parameters as the response rate, the energy consumption or the accuracy of the data acquired. In view of this limitation, it seems that there is a clear opportunity for the development of systems that improve the flexibility of monitoring procedures in this young and very promising research area.

## 3. Proposed Monitoring Platform

Taking into account the importance of the context during the daily life affective assessment, the advantages of mobile technologies for real-time data acquisition, and the lack of flexibility of the existing systems for mood and context monitoring, this work presents a context-aware platform intended to monitor and model the evolution of affective states within naturalistic environments, in a holistic and continuous way. This platform leverages the capabilities of mobile technologies, dealing with the gathering and processing of the sensory data. The system makes use of the sensors available in the smartphones to capture objective context data, as well as self-reports of the affective state, with no need for user interaction with the app further than the response of the questionnaires that are triggered. It also extends the implementation beyond a simple mobile app, integrating a complete architecture for managing and modifying the system configuration during the data acquisition process. By using this technology, we meet the key methodological prerequisites for momentary assessment of affective states proposed in [25]: the ESM affective data is captured instantaneously rather than based on a retrospective recall at the end of the day or week; and the response time is accurately recorded, to ensure that there is no back-filling of answers just before the end of the study. The data gathered is then sent to a secure server where context features can be extracted based on the sensors’ raw data, and used along with the mood data to model the variability of the affective states. In the following, the key features of the system are thoroughly described.

### 3.1. Platform Architecture

In order to achieve the aforementioned functionalities, the platform relies on AWARE, an Android-based open-source context instrumentation framework [26]. AWARE, released under the Apache Software License 2.0 and available at [27], provides a client-server mobile framework that supports the collection of unobtrusive passive sensor data from the smartphone. It follows a modular approach: the AWARE client app enables and abstracts the acquisition of data from the sensors and the communication with the server; then, customizable code extensions called *plugins* can be added to the client, to manage the sensors’ acquisition process, or to generate context information from the raw data gathered. On the other side, the server stores the data and provides an interface to manage the devices connected and the development of research studies. Our system makes use of AWARE to manage the connection and configuration of the devices, the sensor data acquisition and the delivery of ESM questionnaires.

**Figure 2:**
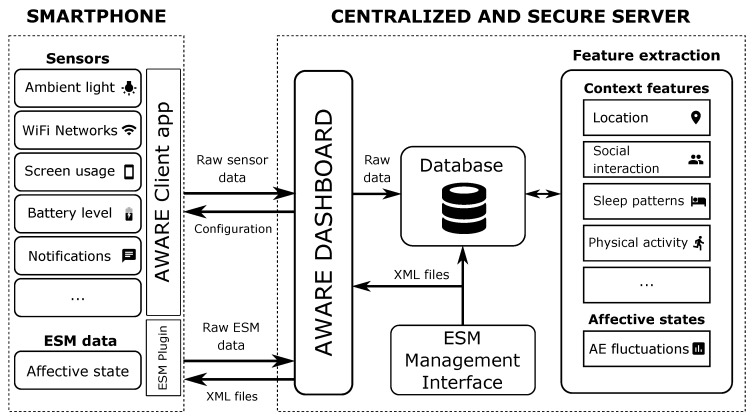
Architecture of the monitoring platform.

Figure 2 shows the architecture of the system. The core entity is the smartphone, which communicates the user and the server. The mobile device uses the AWARE client app to gather the context raw data from the sensors and to sent it to the server through periodic synchronizations. The affective data acquisition is performed using the ESM technique. On the server side, two main elements are deployed. The first of them is the ESM Management Interface, a web-based platform that allows the specialists to configure the settings of the ESM questionnaires to measure the affective state -number of questions, content, response format, scheduling, etc. The interface generates an XML file for each ESM configuration, which is stored in the server. The second element is the AWARE Dashboard, another web platform where the experts can set up the configuration of the smartphone sensors and specify which ESM configuration file is going to be used. The users link their devices to the server by scanning a QR code with the AWARE client app. Then, the ESM configuration file and the sensor setup are received and the app is automatically updated with the new local configuration, making the process transparent to the user. Within the user’s smartphone, the ESM configuration file is read by an AWARE client plugin, especially developed for flexible ESM questionnaire management (see Section 3.3). Once the smartphone is linked to the server, the configuration of the ESMs can be modified at any time using the aforementioned ESM Management Interface. Once the client is configured, the data collection is automatically enabled, and the client stores it locally in a SQLite database [28] deployed on the mobile phone storage unit. This database is cleaned when the data is sent to the server and stored in a central MySQL database, in order to avoid excessive storage consumption. In this way, even if the user loses the internet connection, data continues being stored in the local database, in order to be synchronized with the server when the connection is recovered. In order to preserve the privacy of the data, when the AWARE client app is installed on a smartphone, a randomly generated 128-bit Unique User ID (UUID) is generated—and reset every time that the user scans a different QR code. This way the platform does not collect any personal identifier in its data, and every sample acquired—either by the sensors or by the ESM questionnaires—is only labeled using this UUID. Furthermore, the AWARE framework offers data encryption by default, in order to safeguard the communication between the local and the server database, thus meeting the requirements of the European General Data Protection Regulation (GDPR).

### 3.2. Ecological Momentary Assessment of Affective States

**Figure 3:**
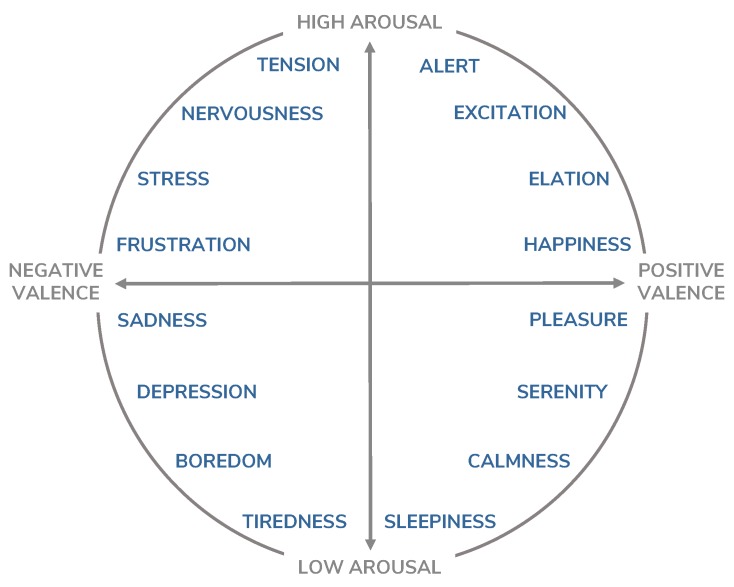
Russell’s circumplex model of mood [14].

Measuring the affective states of an individual is a complicated and subjective task. The affective science has proposed and validated numerous models to measure and describe affect. One commonly used model is the Positive and Negative Affect Schedule (PANAS) [29,30]. This model, based on the idea that negative and positive feelings can be overlapped at the same time, tracks both affects separately. It uses a checklist, often larger than 20 items, to measure the affective states. Despite its accuracy, its extension and complexity make it difficult to use in longitudinal studies where multiple samples are taken in a short period of time. Yet another extended model is the use of discrete categories [31]. This approach requires the evaluation of a number of individual affects, such as happiness, sadness, fear or anger. It is an intuitive and user-friendly approach, but fails to cover a full range of affects. Another available approach commonly used in psychology is the Russell’s circumplex model of mood [32]. This model crosses two dimensions to characterize the affects: valence and arousal. The valence dimension measures the self-perceived pleasure of the mood that the individual is feeling, ranging from highly unpleasant to highly pleasant. The arousal dimension is the self-perceived level of activation (whether the individual is likely to take an action under the mood state), ranging from low activation (calmness), to high activation. Once both dimensions are evaluated, the affective states can be constructed as a combination of both (Figure 3). In this study, we have opted for the use of the circumplex model, due to its simplicity, speed of response and the wide range of mood states that is able to describe. That way it can be measured multiple times per day without taking extensive time from the subjects.

**Figure 4:**
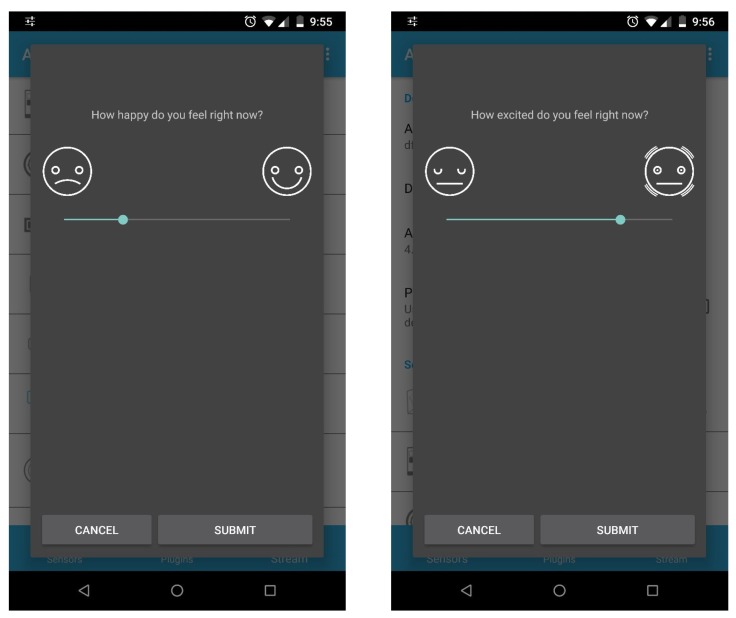
Screenshots of the ESM questions for assessing valence and arousal.

In order to continuously monitor the affective states, individuals have to evaluate the two aforementioned affect dimensions repeatedly over time. To that end, our system takes advantage of the Experience Sampling Method (ESM), also known as Ecological Momentary Assessment (EMA), to prompt a questionnaire on the individual’s mobile phone at a desired time. The questionnaire asks for the input of the self-perceived value of both affect dimensions. Its usefulness beyond traditional paper-based questionnaires for real-time assessment has been successfully explored in recent works [33]. Currently, the ESM questionnaire of this work includes two questions to measure the affect dimensions following the circumplex model: valence and arousal. Traditionally, these dimensions have been measured using a Likert scale [9]. However, given the potential of the mobile technologies, we use a 100-point bipolar scale which is selected using a slider bar. The ESM feature of the AWARE framework allows for the creation of slider-based ESMs. However, we have added extra features to this response format in order to adapt it to the affect monitoring. Instead of showing the numerical value of the scale, we place a simple face icon at each extreme of the slider, whose expressions represent the maximum and minimum values of the scale (Figure 4). For example, in the valence question, the minimum value is represented with a sad face and the maximum one, with a happy face; the arousal question depicts a calm face and an excited face, respectively. Emoticon-based questions have been proved to be more intuitive and user-friendly than numeric based ones [34]. Moreover, although the value of the scale ranges from 0 to 100, it can be interpreted as a bipolar negative-positive scale, clearly communicating the bipolarity to the respondents [35]. Finally, since the ESM is triggered several times per day, the user can get used to the same response if the slider always starts at the same point like the middle or the beginning. To avoid that, a setting has been added to set a random starting point of the slider. A limited set of values can also be specified, and the slider will randomly start from one of these points.

### 3.3. Flexible ESM Questionnaire Management

As it was outlined in Section 2, the existing systems for monitoring affective states and context are limited to a fixed operating mode, especially regarding the experience sampling questionnaires used to capture the affective states. Their methodology—the number of questions, their order, the content of them, the response format and the scheduling—is pre-defined when the application is installed on the smartphone. During the course of an experiment, it may be necessary to modify the content or schedule of the ESM questionnaires, or even sending extra questions in order to maximize the completion rate, capture extra information or keep up the engagement of the participants. For example, rewording the questions or changing their order can increase the response rate and the answer quality, as novel or unfamiliar content has been proved to increase the participant’s engagement [36,37,38] and to mitigate anchoring [39] during longitudinal studies. Some studies have also shown that sending reminders or motivational messages during the course of the study can prevent the engagement level from dropping [40,41]. Extra questions can also be delivered after a period of non-answered questions, asking the participant to recall its affective state during the previous time. In order to achieve this level of flexibility, the proposed platform relies on an architecture designed to enable the adaptation of the data collection methodology during the course of a study. Although the AWARE framework supports basic ESM, we have extended this functionality through the ESM Management Interface, which allows for the definition of the ESM methodology; and a new AWARE Flexible ESM plugin, which configures the ESM questionnaires on the AWARE client app based on the methodology defined. The basic AWARE client app needs the ESM questionnaires to be configured within the source code before compiling the app. By means of the aforementioned tools, the questionnaires can be constructed and changed without recompiling and reinstalling the app. Each ESM question has several configurable properties that define its content and schedule, shown in Table 1. They are defined based on a definition following the XML schema. This architecture ensures a quick intervention in case of a decay in the response rate, a key point for achieving a successful re-engagement [42].

**Figure 5:**
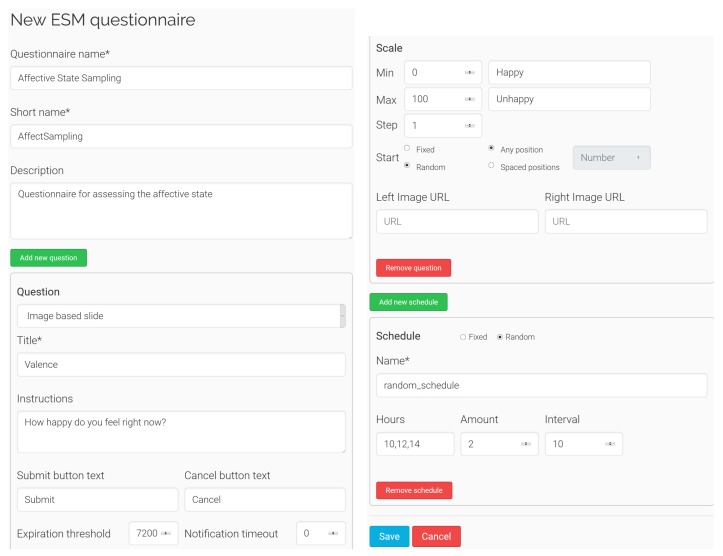
ESM Management Interface.

First of all, the configuration of the ESM questionnaire is defined by the experts using the ESM Management Interface deployed on the server (Figure 5). This web-based interface has been developed using Flask [43], a Python-based web framework, and implements an HTML form which allows for the creation of a questionnaire. Several questions and schedules can be added by clicking the *Add new question* and *Add new schedule* buttons, and removed at any time. All the properties shown in Table 1 can be set to each question and schedule—however, they have default values. The form fields change dynamically if some settings are selected—e.g., changing the schedule type from fixed to random with the corresponding radio button. A different content, response format, and schedule can be set to each question, offering full flexibility to construct the questionnaires. Once the form has been submitted, a configuration file is generated with all the questionnaire settings in XML format. This file is stored in the server, and can be edited at any time using the ESM Management Interface.

Once the questionnaire has been configured, the AWARE client app settings are defined using the AWARE Dashboard deployed on the server. There, the expert can select the sensors which are going to be activated and their sampling rate, as well as the ESM configuration file that is going to be used for the questionnaires. Multiple configurations—named *studies*—can be defined and stored in the dashboard, and a QR code is generated for each study. In order to join the study, the participants simply need to scan the QR code using the AWARE client app, and all the settings are automatically downloaded and configured. Regarding the ESM configuration, we have developed a new AWARE Flexible ESM plugin [44] based on the structure proposed in [33]. This plugin, along with some changes made to the AWARE Client source code, enables the construction of the ESM questionnaires with customized response styles, using the previously generated XML configuration files. When the participant joins the study, the XML file for the ESM questionnaire is also downloaded, and the plugin deserializes it using the Simple-XML serialization framework for Java [45]. All the ESM questions defined in the file are constructed, configured and scheduled in the client app, and the configuration of both ESM content and schedule are stored in the local database, and triggered at the defined time(s).

When the client app has been configured, the participant does not need further interaction with it, making the data acquisition process transparent. As depicted in Table 1, the ESMs can be scheduled either at a fixed time or at a random time within an interval. When the ESM is triggered, a push notification is shown in the smartphone, which the participant has to click to start the questionnaire. The notification persists the amount of time defined by the *NotificationTimeout* property, time after which the notification disappears, thus ensuring that ESM overlapping is avoided. Once the question is answered or canceled, the data of the response is automatically retrieved and stored. The following data is captured: device ID, ESM status -answered, canceled or expired-, trigger timestamp, answer timestamp, initial value of the scale, final value of the answer and completion time of each question. The data is stored within the local database and periodically synchronized to the server. The experts can visualize the amount of data gathered each day for each participant using the AWARE Dashboard. If, based on this information, any modification of the ESM configuration is needed, the experts can modify the configuration files using the ESM Management Interface, which retrieves the data of the XML file and allows for its edition. The AWARE client app periodically downloads the configuration defined on the AWARE Dashboard and checks for any changes. If the ESM configuration file has been modified, the client app automatically updates the ESM configuration, and the subsequent ESM questionnaires will use the new settings.

A common problem found, specially with the last versions of Android, is the presence of battery and memory optimization systems in the operative system of the smartphones. These systems check the applications that are less used and kill their background services, so that the battery and memory consumption is reduced. However, even disabling these systems, some of the ESMs cannot be triggered on the exact time that is defined, because the AWARE service is constantly killed. To solve this problem, the AWARE Client core has been modified to include a method that checks for past schedules that have not been triggered, and triggers them despite the possible small delay existent—usually in the order of a few minutes. The same approach is applied to the server synchronization service.

### 3.4. Automated Monitoring of Context

The context surrounding us refers to any information that can be used to characterize our situation [46]. The objective context acquisition process can be structured in three layers according to [47,48]. The lower layer comprises the raw data directly gathered by the sensing devices. In the middle layer, this data is transformed into context features which provide knowledge about the situation of the monitored individual. Finally, this features are combined in the top layer to infer behavioral markers, which could be considered as the higher-level features. Taking advantage of the amount of sensors available in the smartphones, our system implements the two lower layers of this architecture. The first one is deployed on the user’s smartphone through the AWARE client app. It enables the acquisition of location data (GPS and network), ambient sound and light levels(microphone and light sensor), phone usage data (screen state, battery drainage, notifications, foreground apps, etc.), and movement data (accelerometer and gyroscope). This raw data is automatically acquired and stored in the smartphone, which sends the data to the server through periodic synchronizations of the database. The sampling frequency of all the sensors and the frequency of the database synchronization can be configured using the AWARE Dashboard deployed on the server, which also allows for the tracking of the amount of data gathered from each user. The second layer is implemented on the server. The raw context data is pre-processed to clean it, and combined to build context features. For example, GPS and WiFi data can be used to infer the location type (indoors, outdoors, home, work, etc.), and the ambient light, ambient sound, screen state and battery drainage of the smartphone can be merged to infer the sleep patterns.

## 4. Evaluation

In order to show the potential of the proposed system, and analyze its validity and usability, a preliminary study has been conducted. The aim of this study is to determine whether the data gathered using this system is representative of the participants’ daily life, and therefore, to check its suitability for a real-time context-aware affective state assessment task. The perception and user-friendliness of the system from the end-users was also assessed. Furthermore, the results lead to some recommendations for future studies with similar approaches. In the following, the details and results of the pilot study are described.

### 4.1. Participants

A total of 22 participants—11 males, 13 females, 17–52 years old, M = 22.2, SD = 7.4, were recruited for the study. All of them were required to have an Android-based smartphone and use their own device for the study. Following the ethics approval from the Ethical Committee of the University of Granada, before conducting the study, all the participants were informed about the aims of the research, and they read and signed an informed consent form. Participants were instructed about the installation of the app and the procedure to answer the ESM questionnaires, but no training session or additional information about the use of the app was given, since they do not even need to be aware of the presence of the app, as the data gathering and the ESM delivery is automatic. They were asked to follow the instructions for the installation of the AWARE Client app -including the Flexible ESM plugin-, and the scanning of the QR code for the automatic configuration. Thereafter, they were asked just to continue with their normal lives and answer the ESM questionnaires when received. Three psychology experts designed the ESM questionnaire and supervised the study.

### 4.2. Methods

#### 4.2.1. Procedure

In the first place, the three experts designed the ESM questionnaire for assessing the affective states described in Section 3.2, by means of the ESM Management Interface. After that, the app installation and configuration procedure was carried out on the participants’ smartphones. We collected data for a total of 14 consecutive days, including both weekdays and weekends. ESM-based studies are recommended to have a duration of 2–4 weeks, because response rate and accuracy is proved to drop after this time due to fatigue [13,49,50]. During this period, both affective states and validity indicators were monitored. There is no agreement in the literature on the ideal number of notifications, usually ranging from 1 to 10 per day [16]. However, it is suggested to gather the minimum required number of samples for having valid data, without cluttering participants [13,50,51]. In our study, six questionnaires were delivered per day at pseudo-random times. The schedule of the notifications must also be taken into consideration. Researchers are encouraged to use random or pseudo-random schedules [19], because they reduce the chance of biased reports. Nevertheless, it is necessary to ensure that the samples are not too close, which could produce redundant information. For that reason, we set six evenly distributed intervals of one hour, during which the questionnaire can be triggered. The intervals selected were: 07:00–08:00, 10:00–11:00, 13:00–14:00, 16:00–17:00, 19:00–20:00 and 22:00–23:00. Upon receiving the questionnaire, participants had 120 min to open the notification before it disappears and is marked as *expired*. The two questions are presented sequentially: once the valence level is reported, the arousal question appears on the screen. Finally, the penultimate day of the study, a reminder was sent as an extra ESM using th ESM Management Interface, in order to notify the participants of the upcoming end of the study and give them the instructions to stop the application.

#### 4.2.2. Validity Indicators

In order to assess the validity of the affective state measurements, we have calculated a number of context indicators regarding the ESM questionnaires. These features have been used in previous works [49,50,52] as indicators of the validity of the data acquired. The validity indicators are measured with regard to each questionnaire: hour of the day and relative day of the study when the questionnaire was responded; completion time for the questionnaire; and time elapsed between the questionnaire reception and its completion. The response rate has also been assessed.

#### 4.2.3. Usability Evaluation

The goal of the last part of this evaluation is to assess the usability and interest of the proposed system, according to the users’ and experts’ opinions. To that end, we employed the System Usability Scale (SUS) [53]. This scale has become an industry standard used to quantify user’s experience with a system. The SUS consists of a 10-items questionnaire, with every answer scored by the user through a 5-point scale ranging from “strongly disagree” to “strongly agree”. It is a quick and reliable method that provides an indicator of the usability of the system, and has been tested on a wide variety of systems [54]. The SUS questions are the following:

(Q1) I think that I would like to use this system frequently.
(Q2) I found the system unnecessarily complex.
(Q3) I thought the system was easy to use.
(Q4) I think that I would need the support of a technical person to be able to use this system.
(Q5) I found the various functions in this system were well integrated.
(Q6) I thought there was too much inconsistency in this system.
(Q7) I would imagine that most people would learn to use this system very quickly.
(Q8) I found the system very cumbersome to use.
(Q9) I felt very confident using the system.
(Q10) I needed to learn a lot of things before I could get going with this system.

At the end of the study, all the participants were asked to fill a survey with these questions regarding the use of the AWARE client app with the Flexible ESM plugin, as well as to provide feedback on their use if desired. In the same way, after using the ESM Management Interface, the three experts were asked to provide their impressions regarding its use, and to evaluate it with the System Usability Scale.

### 4.3. Results

#### 4.3.1. Response Rate

**Figure 6:**
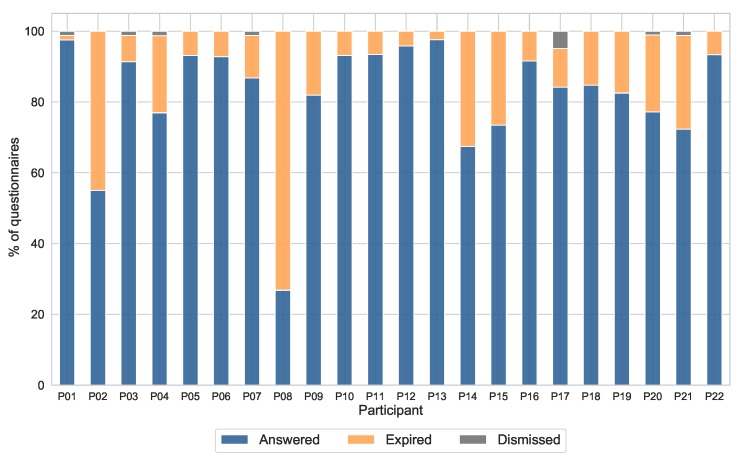
Percentage of questionnaires answered (blue), expired (orange) and actively dismissed (grey) for each participant during the entire study.

The response rate is an indicator of how well the data acquired represent the real feature studied. For that reason, it can be used to assess the validity of the proposed system towards the monitoring of affective states. During the two-week data collection period, a total of 1848 questionnaires were triggered. Participants completed a total of 1369 questionnaires, which means an overall response rate of 74.1%. A total of 11 notifications were actively dismissed by the participants (0.6%), while the 468 remaining questionnaires (25.3%) were automatically dismissed after 120 min. However, this feature also has to be measured individually, in order to evaluate whether the amount of valid data acquired for a particular subject is enough to represent correctly the evolution of the affective states. Figure 6 shows the percentage of answered, dismissed and expired questionnaires for each subject. It can be seen that the individual response rate ranges from 27.8% (subject P08) to 97.6% (subject P13), with a mean value of 82.2% (±16.5%).

**Figure 7:**
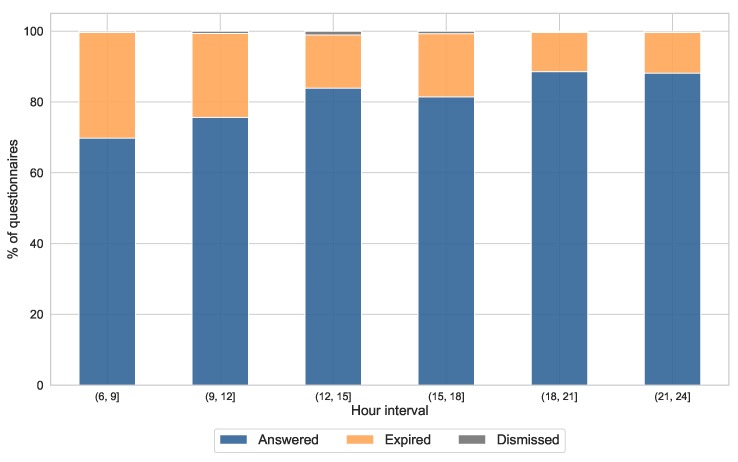
Overall percentage of questionnaires answered (blue), expired (orange) and actively dismissed (grey) per interval of daily hours.

In our study, we triggered six questionnaires at different times during the course of the day. The response rate varies depending on the time when the questionnaire is submitted. Figure 7 depicts the overall response rate in our study, versus the hour of the day when the ESM notification was triggered. The ESM schedule was randomly set within one hour intervals, so the hours in this figure are grouped within intervals evenly spaced around the triggering intervals. The response rate was considerably lower in the first half of the day between 06:00 and 12:00, increasing as the day progresses. The highest response rate is achieved between 18:00 and 00:00, with a value of 88.1%, which means an increase of a 18.3% with respect to the beginning of the day.

**Figure 8:**
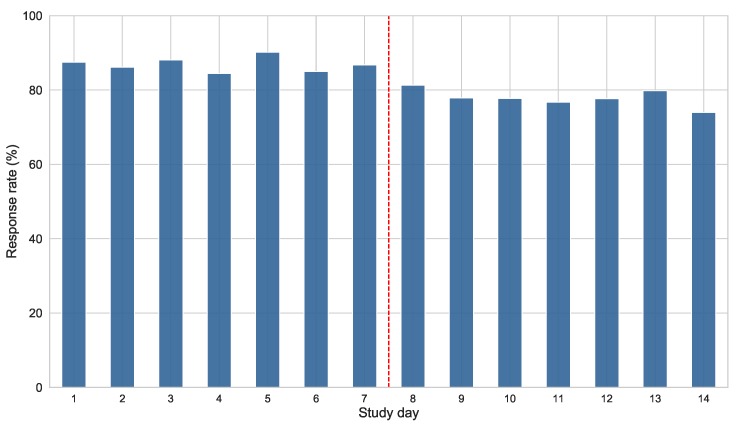
Overall response rate registered per day of study. The red dashed vertical line splits the graphic in the two weeks of the study.

The response rate also fluctuates during the course of the study. Figure 8 shows the evolution of the overall response rate as the study goes on. The central vertical line emphasizes the distinction between the two weeks covered by the study. It can be seen that the response rate keeps significantly high during the first week of the study, experiencing a progressive decrease in the second week. The mean response rates for each week are 86.9% and 77.9%, respectively. The previously mentioned reminder of the end of the study was sent on day 13. This reminder could explain the slight increase of responses appreciated in this day, as the participants started again to keep the questionnaires in mind.

#### 4.3.2. Completion Time

**Figure 9:**
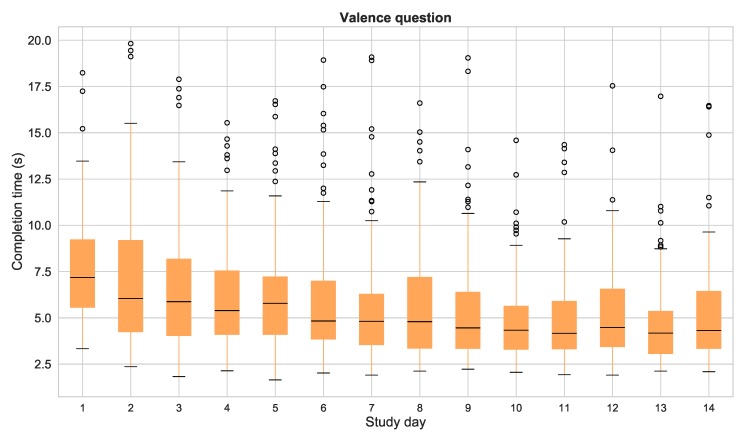
Completion times of the questionnaires per day of study for the valence question. Times over 300 s have not been considered.

**Figure 10:**
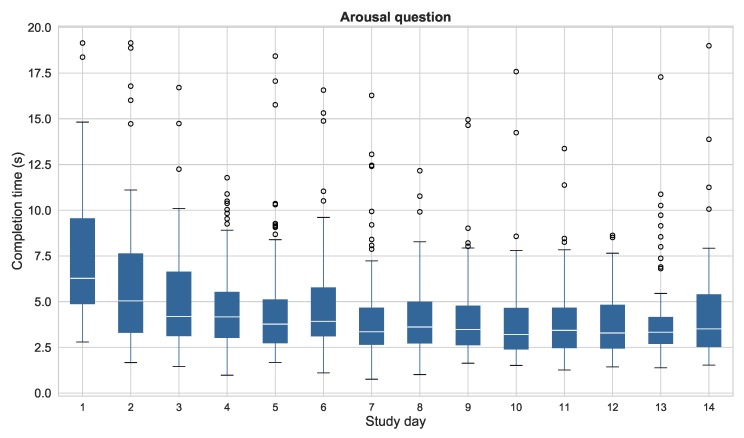
Completion times of the questionnaires per day of study for the arousal question. Times over 300 s have not been considered.

The time elapsed between the opening of the notification and the submission of the answer has also been measured for this analysis. It is an indicator of the attention paid while responding of the question. The mean (±standard deviation) completion time for all the questionnaires was 7 (±7.54) and 4.9 (±4.2) seconds for the valence and arousal questions respectively. These values include the time spent by the plugin to load all the resources of the questionnaire including the images. Following the recommendations of [49], we have removed the responses with completion times two standard deviations above the mean, since they may be caused by problems experienced when loading their resources or by excessive inattention during the response, thus producing low quality data. Figure 9 and Figure 10 show the mean completion times for each study day for both valence and arousal questions. This analysis shows two important results: first of all, it is worth mentioning that the mean completion times of the valence question are substantially higher than the ones for the arousal question, presumably as a result of the order of presentation of both questions (valence was the first dimension to be assessed). Secondly, the completion time considerably decreases during the first week of the study, and remains almost constant during the second week. This behavior is shared by both questions.

#### 4.3.3. Elapsed Time from Notification Arrival to Response

**Figure 11:**
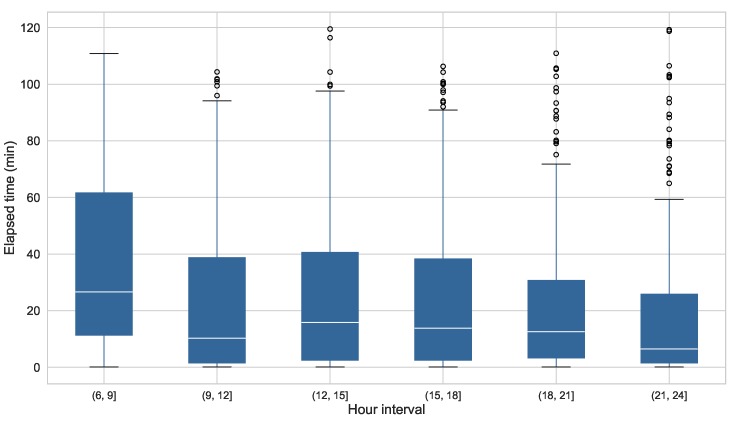
Time elapsed from the reception of the notification to the participant’s response per interval of daily hours.

For this evaluation, the time elapsed between the arrival of the notification to the time when the participant clicks on it and respond the questionnaire has also been measured. As the notification of the ESM persisted for 120 min after its reception, they could be responded out of the triggering intervals, thus modifying the time distribution of the samples. Computing the elapsed time from the notification to the response, we can get an idea on when the participants actually answer the questionnaires. It should be taken in consideration that this analysis only includes the questions that have been responded or dismissed, since the ones that expired do not have response time. Figure 11 depicts the elapsed times versus the hour of the day when the notification was triggered.

It can be observed that in the early morning interval, from 06:00 to 09:00, participants take more time to open the questionnaire, with a mean (±standard deviation) elapsed time of 37.2 (±32.1) min. This value is considerably lower during the rest of the day, reaching its minimum point at the end of the day, from 21:00 to 00:00, with a mean elapsed time of 18.3 (±25.9) min.

#### 4.3.4. Usability Assessment

A total of 20 of the 22 participants of the study completed the usability survey about the app at the end of the experiment, and the three experts did the same for the ESM Management Interface. They rated the 10 items stated in Section 4.2 using a Likert scale ranging from 1 or “strongly disagree” to 5 or “strongly agree”. In order to compute the global SUS score for each user, an individual score is given to each item, following the guidelines presented in [53]:

1. For odd-numbered items, the score is computed subtracting 1 to the user response.
2. For even-numbered items, the score is computed subtracting the user response to 5.
3. All the scores obtained—now ranging from 0 to 4, are added and multiplied by 2.5 to obtain the overall SUS score.

**Figure 12:**
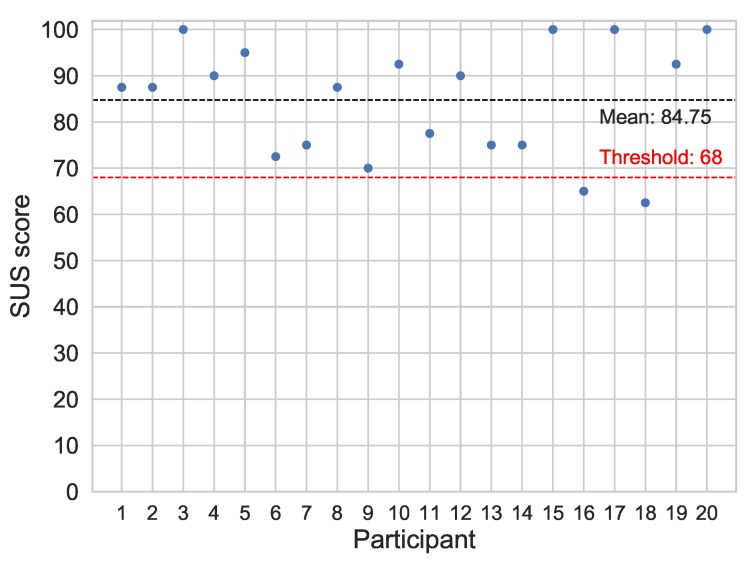
System Usability Scale (SUS) score obtained from each participant. The horizontal lines represent the mean SUS score of the system and the threshold value that indicates a good usability.

The average standardized SUS score is 68 [54,55]. A system with a score over this value is considered to have a good usability level. Moreover, systems who exceed a SUS score of 80.3 are considered to have an excellent usability level. Regarding the app and the plugin, the SUS scores given by each participant of the study after computing their overall value are shown in Figure 12. The black dashed line indicates the mean value of the SUS scores given to our system, and the red one indicates the aforementioned good usability threshold. It can be seen that only two out of the twenty ratings are under 68, and the mean SUS score given to the system is 84.75. These values represent high levels of acceptability and ease of use, thus indicating that the usability of the system seems to be highly favorable for the end users.

With the aim of complementing this evaluation and have a better understanding about the ratings of the system, we also asked the participants to give voluntary feedback about the performance of the system. First, most of them noted the ease of use of the ESM questionnaires, and remarked that the response to them was much less time consuming than they expected: “*The application is very intuitive, it automatically launches the questionnaires and you can answer very easily and quickly.*” (P15). Some of them also gave some indications about the procedure of responding the questionnaires, pointing that both affective dimensions (valence and arousal) were difficult to define in such a wide scale, specially the arousal one. They suggested that it could be easier to evaluate using a scale with more restricted values (e.g., a Likert scale). They also commented that, although the face icons helped to identify the feelings at the beginning, after one or two days responding the questionnaires, they go unnoticed. One participant also suggested that “*It could be interesting to access to the questionnaire directly when unlocking the phone.*” (P02). Although the participants did not report special negative comments, some of them noted a slight increase of the battery drainage during the course of the day: “*The only negative point could be that the battery drainage is noticeable even when the phone is not being used.*” (P18). Despite of this fact, almost all the participants noticed that the extra battery drainage still allowed a normal daily operation of the smartphone.

With respect to the ESM Management Interface, the three experts gave a SUS score of 92.5, 87.5 and 95, respectively, resulting in a mean SUS score of 91.67. This score shows an excellent level of usability and acceptability of the system among the experts. They also provided their impressions regarding the use of this tool. They appreciated its high level of customizable settings and its easiness of use, since it makes very straightforward the process of configuring the questionnaires: “*The tool offers several options and is very intuitive and easy to use, since it guides the process so that the main issues are addressed.*” Likewise, the experts were truly impressed with the easiness to make modifications of the already defined questionnaires. The only negative issue reported is that the aspect of the ESM Management Interface when visualized in mobile devices is not confortable, since the aspect ratio of the elements does not suit properly the smartphone screen. However, despite this positive evaluation, to further confirm the usability of this tool, a study with a higher number of experts is needed, but these preliminary findings seem to be favorable.

## 5. Discussion

In this paper, we presented a smartphone-based platform intended to monitor the affective states and the context of an individual, with a flexible management of the data acquisition process. We conducted a pilot study to show the potential of the platform and assess its validity, as well as the usability of its elements. The results obtained and the implications and recommendations for future studies using this technology are further discussed in this section.

**Response rate**. The response rate is an indicator of how well does the data acquired represent the real feature studied. A high response rate indicates that the affective states have been captured in a wider variety of scenarios, so that it is more likely to be contextually diverse. In the opposite situation, a low response rate means a lack of samples, making it more difficult to represent faithfully the affective state fluctuations. Although there is no agreed gold standard for acceptable response rate, some studies noted that ESM data requires a compliance rate close to 80% to be representative of participants’ daily life [16,50]. Some researchers do sometimes remove from the analysis those participants with low compliance rates, as their no-representative results can bias the general analysis. The response rate of 17 out of 22 participants of our study are above 77%, verifying the validity of the data gathered and, thus, showing that the proposed system is suitable for monitoring the affective states through ESM questionnaires. However, this marker also shows that there are three participants that should not be considered in further analyses (P02, P08 and P14), because of having compliance rates of 55%, 26.8% and 67.4% respectively.

The response rate also varied within day and within the course of the study. We found that the response rate was considerably lower during the early hours of the morning (06:00 to 12:00), experiencing an increase during the day and reaching its maximum level at the last hours (18:00 to 24:00). This result was expected, because schedules are usually busier during the morning, and the participants might find themselves in situations such as work, lectures, or other environments where they are not aware of the mobile phone, or free to use it. To overcome this issue, it could be interesting to include some recall questionnaires at particular times during the day or to modify the schedules to fit the participants schedule, using the ESM Management Interface Regarding to the day of the study, several researchers warn of a decline in ESM data quality linked to the study duration. In [50], the recommended duration for experience sampling studies is between 2–4 weeks. In [49], a considerable drop in the response quality was found in the third week of study. However, it is advisable to keep a minimum duration of one week in order to ensure enough contextual variety in the data captured [13]. In our study, we found a considerable drop in the number of responded questions from the second week of study (9% of decrease with respect to the first week). However, the daily response rate during the second week remains close to 80%, so we can consider that these data is still representative of the participants’ daily life. In fact, we noticed a slight increase in the response rate one day before the end of the study. This day, a reminder was sent to the participants using the ESM Management Interface with the instructions to end the data collection on the next day. The increase in the response rate may be related to this reminder. Based on this finding, we can both confirm the good performance of the ESM Management Interface, and set up an approach for keeping a high engagement level by sending periodic reminders.

**Completion time**. When the participants of a study have to answer the same questionnaire several times, they get used to the questions and the response format, and gradually “automatize” the response. The literature links extremely low completion times to inattention when responding the questionnaires, and several studies recommend removing questionnaires with suspiciously fast completion times [13,52]. In our study, the responses with completion times two standard deviations above and below the mean have been removed according to [49]. Less than the 5% of the questions are outside these limits, so the remaining data is still enough to keep a high response rate. We also find that the first day, the mean completion time is substantially higher than the rest of the study, presumably due to the novelty of the questionnaires and the difficulty to identify correctly the valence and arousal levels. The daily mean completion time in our study decreases during the course of the study, specially during the first week. During the second week, the value remains constant. This decrease could be a result of a the aforementioned habituation effect. This automation process can be dangerous, since the users can pay less attention to their responses and the data quality could decrease. In that case, reformulating the questions or changing the response format during the course of the study, using the ESM Management Interface could help to maintain a high attention level when responding to the questions. A suggestion intended to keep the completion time more constant is to perform a learning phase at the beginning of the study, during which the participants are taught to identify correctly their valence and arousal levels, and get used to the procedure of responding the questionnaire, thus avoiding the initial higher completion times.

It is also worth mentioning the difference in the completion time of the valence question and the arousal question. The arousal question was responded an average of three seconds faster than the valence one. Further analyses are required to determine if this finding results from an easier identification of the arousal level, or from the order of presentation of the questions, since the valence question was presented first and participants may take more time to start the response procedure.

**Elapsed time from notification arrival to response**. The analysis of this measurement show that, during the early hours of the day, participants wait more time from the reception of the notification to the response of the questionnaire. This finding reinforces the previous affirmations about the response rate, which was lower during this period of the day. The implications of this finding for future studies are that, even when the participants respond to the questionnaires during the mornings, the sampling times could not be evenly spaced, so the data gathered through mobile ESMs during the morning may have less quality. Further analysis will be needed to determine, based on the context captured, which are the most suitable locations or moments to send an ESM questionnaire during the morning, thus considering to modify the schedule during the study to fit the participants’ daily routine.

**Usability**. The results of the usability analysis show that the proposed platform has a high level of user-friendliness, acceptability and ease of use, both from the participants’ and the experts’ perspective. The mean SUS scores given to the app and the ESM Management Interface were 84.75 and 91.67, respectively. It proves that the use of the platform does not require technical skills, making it appropriate for its spread use among the population and the scientific community. The feedback of the users and the experts has been highly valuable, and has let some suggestions for an improvement of the platform. For example, it could be interesting to open the questionnaires upon unlocking the phone, thus making it simpler and faster for the users to respond. It may also increase the response rate due to this more faster approach. This *smartphone-unlock* triggering has been explored in other studies, with an increase in the response rate [56]. Finally, if more sensors are intended to be activated in future studies, the battery drainage should be optimized to ensure a normal daily routine of the subjects, in order to keep the data as much reliable as possible, avoiding any type of intervention. The ESM Management Interface has had a great acceptability among the pysichology experts that supervised the study, who praised its usefulness for exploring new approaches of interventions during the course of research studies. According to the experts comments, it should be optimized for a better visualization in mobile devices.

**Limitations**. Finally, we discuss some limitations of the study that could have affected our results. First of all, the age group of the participants was limited, with a mean age of 22 years old. It biases the type of situations in which the participants are involved during their daily life. For example, a great part of the participants attended to lectures or work during the mornings, thus not being able to respond the questionnaires. A more wide range of ages would be recommended for future studies. Also could be interesting to conduct the study during a longer time frame, for a further confirmation of the results obtained. The platform should also be assessed by a higher number of experts to further confirm the usability levels reached in this study.

## 6. Conclusions

The continuous monitoring of the fluctuation of the affective states over time is a challenging, but essential task in emotion research, as the changes in our mood strongly influence our cognition and social behavior. In order to identify the daily life events that trigger significant changes on our affective states, this task often requires to be accompanied by the monitoring of the context surrounding the individual. This work finishes the implementation of an early prototype proposed in [14] and presents a smartphone-based platform intended to perform a ubiquitous, context-aware monitoring of the affective states during the daily life. The platform provides a complete working system to measure the context surrounding the user through the sensors available in the smartphone, and to take advantage of the ESM technique to monitor the affective states of the user through self-reports. The system integrates the data gathering app with a web interface intended to easily configure the content and schedule of the ESM questionnaires, allowing for their modification during the data acquisition process. All the data gathered is stored in a centralized server, where context features can be extracted from the raw data gathered by the smartphone. The platform is intended to provide an easy method for acquiring context-sensitive affective data and to give a high level of flexibility to the ESM questionnaire management. In order to show the potential of the platform and assess its usability, a two-week pilot study has been conducted, gathering affective and context data from 22 users. The results show that the response rate and the completion time of the ESM questionnaires reach the standard values required to confirm the suitability of this platform for affective states monitoring. The results also show the potential of the ESM Management Interface for applying interventions during the course of the study, so that the participants’ engagement keeps in a high level. The usability of the system has been assessed through the SUS scale, showing an excellent acceptance level among the participants of the study and also among the experts. Based on our findings, we also offer some recommendations to improve the quality of the data gathered using this platform. These recommendations will also be applied by us in future experiments, where we expect an increase of the response rate and the data quality. Future works include a longer study with a higher number of participants, also monitoring more context features, in order to generalize this findings and identify the events that trigger relevant changes on the affective states.

## Figures and Tables

**Table 1:** Configurable properties of the ESMs within the Affective Flexible ESM plugin.

Property	Description	Type
ESM_Type	Response format of the ESM question	string
Title	Title of the ESM question	string
Instructions	Instructions to answer the ESM question	string
SubmitText	Text of the button to submit the answer	string
Canceltext	Text of the button to cancel the question	string
ExpirationThreshold	Time available for completing the question before it is removed (in seconds)	int
NotificationTimeout	Time that the notification remains in the notification bar (in seconds)	int
ScaleStartRandom *^†^	Enable the initialization of the slider in a random position	boolean
ScaleStartRandomValues *^†^	Enable the initialization of the slider in a random position among a range of values	int
ScaleStart ^†^	Fixed initial position for the slider	int
ScaleStep ^†^	Value increment of each step of the slider	int
ScaleMin ^†^	Minimum value of the scale	int
ScaleMinLabel ^†^	Label displayed for the minimum value of the scale	string
ScaleMax ^†^	Maximum value of the scale	int
ScaleMaxLabel ^†^	Label displayed for the maximum value of the scale	string
LeftImageUrl *^†^	URL of the image displayed on the minimum value of the scale	string
RightImageUrl *^†^	URL of the image displayed on the maximum value of the scale	string
ScheduleTime	Time (HH:MM) on which the ESM is scheduled	datetime
ScheduleRandom	Enable the random scheduling of the ESM within an interval of time	boolean
ScheduleRandomAmount ^‡^	Amount of times that the ESM is scheduled in the interval	int
ScheduleRandomInterval ^‡^	Minimum time left between random ESMs	int

* Extra properties added to the basic AWARE ESM feature. ^†^ Only required if the value of ESM_Type is set to ESM_Scale_Image. ^‡^ Only required if ScheduleRandom is set to true.
